# Rice origin traceability using mid-infrared and fluorescence spectral data fusion

**DOI:** 10.3389/fpls.2025.1679754

**Published:** 2025-11-18

**Authors:** Changming Li, Yong Tan, Chunyu Liu, Xun Gao, Zhong Lv, Hongchen Zhang, Yong Zhang

**Affiliations:** 1 School of Physics, Changchun University of Science and Technology, Changchun, China; 2 Engineering Tech, R&D Center Changchun Guanghua College, Changchun, China

**Keywords:** spectrometry, data preprocessing, origin discrimination, machine learning, data fusion

## Abstract

This study overcomes the limitations of traditional single-spectroscopy techniques by constructing an intelligent discrimination system for rice geographic origin that integrates mid-infrared (MIR) and fluorescence (FLU) spectral feature fusion with machine learning. Using the “Zhongke Fa 5” rice variety from eight major production regions in Jilin Province, China, as the research object, spectral data were acquired using Fourier transform infrared (FTIR) and fluorescence spectrometers. A “Normalization-Smoothing-Multiplicative Scatter Correction” preprocessing framework was proposed, significantly enhancing the signal-to-noise ratio and separability of the spectral features. The complementary characteristics of the multispectral data were elucidated: MIR spectra (500–3750 cm^-1^) accurately represented molecular vibration features of key components such as starch, protein, and lipids, while FLU spectra (450–850 nm) effectively captured the fluorescence characteristics of phenolic compounds and protein-pigment complexes. The successive projections algorithm (SPA) was employed to extract 286–310 highly discriminative features from the original 7625-dimensional data, effectively mitigating the overfitting problem associated with high-dimensional data. The performance differences between data-level and feature-level fusion strategies were compared. The feature-level fusion model optimized by SPA demonstrated significant advantages, achieving a test set accuracy of 95.55%. Regarding algorithm performance, the logistic regression (LR) model combined with enhanced spectral features (LR-SPA) significantly outperformed support vector machine (SVM, 83.17%) and gradient boosting algorithms in terms of both precision (93.05%) and robustness. This study provides a revolutionary technical approach for agricultural product quality and safety supervision, holding substantial theoretical innovation and practical application value. As a primary goal, the abstract should render the general significance and conceptual advance of the work clearly accessible to a broad readership. References should not be cited in the abstract. Leave the Abstract empty if your article does not require one – please see the “Article types” on every Frontiers journal page for full details.

## 1 Introduction

Rice, as one of the world’s most crucial staple crops, has its quality highly scrutinized. Rapid identification of rice origin remains a focal and challenging point for both consumers and researchers. In recent years, spectroscopic techniques have gained widespread recognition and application in the field of agricultural product origin identification due to their non-destructive nature. Spectral information can precisely reflect the composition and content of organic compounds in rice, making it a vital technical means for rice origin traceability. Rice from different regions exhibits internal compositional differences due to multiple factors such as climate, environment, and soil, leading to diverse spectral characteristics.

Compared to the drawbacks of traditional detection methods (e.g., High-Performance Liquid Chromatography, HPLC; Gas Chromatography, GC), such as low detection efficiency, expensive equipment, and complex sample preparation (requiring sample destruction) (Coronel-Reyes et al., 2018; Wang et al., 2019; Men et al., 2020), non-destructive testing techniques are superior due to their high efficiency, accuracy, and potential for real-time detection. It should be noted that although methods like HPLC/GC are time-consuming, their high accuracy and repeatability have been validated by extensive research (Berrueta et al., 2007; Azmi N, Kamarudin et al., 2021; Zhang et al., 2023a) and they remain the gold standard for compositional analysis. Wang M summarized the major technical methods for detecting aromas in agricultural products, with a focused analysis on the advantages and limitations of techniques such as chemical sensors, gas chromatography–mass spectrometry (GC–MS), and electronic noses. The review further explores the application prospects of these technologies in smart agriculture and their integration trends with the Internet of Things (Wang et al., 2024).Traditional rice identification methods (e.g., morphological observation, biochemical detection) are typically labor-intensive, time-consuming, and complex (Gong et al., 2023), unsuitable for batch analysis and non-destructive testing requirements.

Previous studies have extensively explored the application of spectroscopic techniques in agricultural product identification. Specific achievements include: Sheng Gong et al. using MIR spectroscopy and random forest algorithms to trace the origin of medicinal materials (Liu et al., 2024); Bing Liu et al. constructing a Partial Least Squares-Neural Network (PLS-NN) model for efficient and accurate identification of Cornus officinalis origin based on MIR data (Luan et al., 2023); Luan Xinxin et al. effectively discriminating rice origin by combining multiple spectroscopic methods with chemometric analysis (Xiao et al., 2024); Xiao Yuhui et al. proposing spectral augmentation techniques combined with IR spectroscopy and machine learning to improve soybean identification accuracy (Jin et al., 2022); Baichuan Jin et al. employing near-infrared (NIR) hyperspectral technology and various machine learning methods to build a rice seed variety identification model (Lei et al., 2022). Furthermore, Lei Yuanxiong et al. preprocessed transgenic soybean spectral data and established Partial Least Squares - Discriminant Analysis(PLS-DA) and Backpropagation (Artificial) Neural Network(BP-ANN) models, models, finding the BP-ANN model achieved 100% discrimination accuracy (Teye et al., 2019); Teye et al. collected rice spectral data from different origins, established K-Nearest Neighbor models after preprocessing, achieving best recognition rates of 90.84% and 90.64% for the training and prediction sets, respectively (Zhang et al., 2015). These studies have not only achieved discrimination between different rice types (Chen and Huang, 2010; Chen et al., 2018) but also involved rice quality measurement (Li et al., 2016), cultivar classification, and applications in rice authenticity testing (Siriphollakul et al., 2017), protein and starch content prediction, waxy rice detection, and edible quality prediction (Hao et al., 2024).

Data fusion techniques have been widely applied to enhance the accuracy of spectral analysis. Nan Hao et al. successfully identified the origin of Lonicerae japonicae flos using a data fusion strategy combining NIR and MIR spectroscopy (Robert et al., 2021); Robert et al. explored data fusion methods combining Raman and IR spectroscopy for predicting red meat parameters (Dai et al., 2023); Dai et al. studied the use of NIR and Raman spectroscopy to distinguish rice from similar origins (Vitelli et al., 2021); Michael Vitelli et al. utilized multiple spectroscopic techniques combined with data fusion to analyze the main components of potato flour samples (Wang et al., 2023). Additionally, Wang Zhiqiang et al. discussed the application of spectral data fusion technology for rapid detection of rice protein content (Song et al., 2024); while Chenxuan Song et al. developed a novel method for identifying contaminated rice through data fusion of NIR spectroscopy and machine vision (Cui et al., 2007).Recent research also indicates that metabolomics methods based on chromatography-mass spectrometry can provide more refined origin characteristic markers, but their cost and complexity limit large-scale application.

However, constructing universal origin discrimination models still faces challenges such as high sample diversity and numerous background interference factors. It is difficult for models to distinguish whether spectral signals originate from genetic varietal differences, climatic conditions, or specific local environmental factors. Addressing this challenge, this study proposes a new research path: during the initial method development phase, first deeply validate the resolution sensitivity of the traceability technology at a “controlled variable” micro-geographical scale, thereby laying the foundation for large-scale application. Consequently, the core objective of this study is to verify the ultimate resolution capability of the spectral data fusion strategy for rice origin traceability at a micro-geographical scale.

To achieve this goal, we selected eight major production areas within Jilin Province, China, as the study region. Significant gradients in topsoil pH and soil nutrient content exist within the province, while the main planting areas predominantly use the “Zhongke Fa 5” variety. This provides an ideal scenario for precisely parsing the impact of micro-environments on elemental fingerprints while controlling for cultivar variables.

This research focuses on characteristic japonica rice from Jilin Province, integrating MIR and FLU spectroscopy. By systematically optimizing spectral preprocessing methods (including normalization, Savitzky-Golay smoothing, and multiplicative scatter correction) and combining them with machine learning algorithms, an efficient and accurate novel method for origin discrimination was constructed. This study not only provides a reliable traceability tool for Jilin rice but also its methodology—validating model sensitivity through micro-geographical scale case studies—offers a theoretical basis and practical foundation for future expansion of the technical framework to broader geographical regions.

## 2 Experimental section

### 2.1 Samples

#### 2.1.1 Sample source and preparation

Samples were collected during the rice maturity period in mid-to-late September 2024, covering the central and western main rice production areas of Jilin Province. A total of 120 rice samples were obtained from 8 different origins (Dehui DH, Gongzhuling GZL, Huadian HD, Shulan SL, Taoer River TRH, Yitong YT, Yushu YS, Zhenlai ZL). The detailed information on average temperature, accumulated temperature, precipitation, average relative humidity, and climatic characteristics for each production region during May to September 2024 is provided in **Table 1**. Three representative paddy fields were selected from each origin as biological replicates. Five sampling points were set along the diagonal in each field, and the samples were mixed to form one representative sample per field, with each sample weighing approximately 1.0 kg. All samples were from the same batch of the “Zhongke Fa 5” variety to control for the effects of varietal variation.

**Table 1:** Climatic characteristics of production regions including average temperature, accumulated temperature, precipitation, and mean relative humidity.

Production region	Mean temp. (May-Sept) (°C)	Accumulated temp. (°C)	Precipitation (mm)	Mean relative humidity (%)	Climatic characteristics
DH	18.2-22.1	2800-2900	380-420	72-76	Temperate semi-humid climate with synchronous rain and heat periods
GZL	19.0-22.8	2900-3000	400-450	70-74	Black soil region with precipitation concentrated from June to August
HD	17.5-21.3	2700-2800	500-550	78-82	Mountain climate characterized by significant diurnal temperature variation
SL	17.8-21.9	2750-2850	450-500	75-79	Short frost-free period with rapid temperature drop in autumn
TRH	18.5-23.2	2950-3050	300-350	62-66	Semi-arid climate with abundant sunshine
YT	18.8-22.5	2850-2950	420-470	71-75	Sufficient accumulated temperature but high interannual precipitation variability
YS	18.0-22.0	2800-2900	390-440	70-74	Chernozem region prone to strong spring winds
ZL	19.2-23.8	3000-3100	280-330	58-63	Eastern fringe of the Horqin Sandy Land with intense evaporation

Agronomic information obtained through field interviews indicated consistent cultivation management practices across sampling sites: sowing in early May, maturation in late September, irrigation primarily using groundwater, and fertilization rates of 140 kg/ha nitrogen (N), 75 kg/ha phosphorus (P_2_O_5_), and 80kg/ha potassium (K_2_O). Samples were delivered to the laboratory within 24 hours after harvest on October 20, 2024, and stored temporarily under constant temperature conditions.

**Figure 1:**
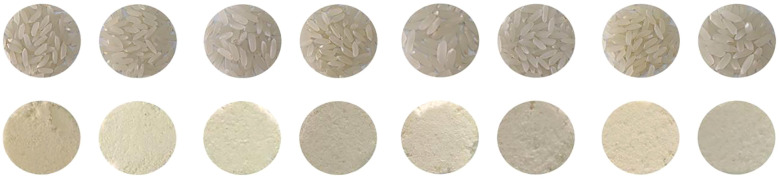
Images of samples to be tested (Rice origins from left to right: Dehui, Gongzhuling, Huadian, Shulan, Taoer River, Yitong, Yushu, Zhenlai).

Sample pretreatment followed a standard procedure: Paddy rice was equilibrated for 72 hours at a temperature of (25 ± 2)°C and relative humidity of (60 ± 5)%. After dehulling using a husker, 100 intact, plump, and disease-free brown rice grains were randomly selected, ground using a dedicated mill, and passed through a 100-mesh (150 μm) metal sieve (according to GB/T 6003.1-2012) to ultimately obtain powdered samples with uniform particle size (**Figure 1**). This treatment effectively reduced spectral measurement errors caused by differences in sample physical properties.

### 2.2 Experimental instruments and data acquisition

#### 2.2.1 Experimental instruments

This experiment utilized two advanced spectroscopic analysis devices for sample detection: an ATP2400 fiber optic spectrometer from Optosky (Xiamen) Photonics Inc. (Xiamen, China), and a Nicolet™ iS50 Fourier Transform Infrared (FTIR) spectrometer from Thermo Fisher Scientific (Waltham, MA, USA). The ATP2400 spectrometer has a broad spectral detection range of 350–800 nm, uses a 405 nm excitation light source, and employs 5 repeated scan averages to improve the signal-to-noise ratio (SNR). Its probe features a vertical detection design, maintaining a fixed 100 mm detection distance and a 20°field of view, connected via an SMA905 standard interface to a UV600-1.0 quartz optical fiber, and is equipped with a USB data transmission interface. The Nicolet™ iS50 FTIR spectrometer utilizes a 24-bit 500 kHz high-speed A/D converter, is equipped with a Polaris™ long-life dual IR source (15-27,000 cm^-1^), and supports adjustable scanning rates from 0.158 to 6.28 cm/s at a high resolution of 0.09 cm^-1^.

#### 2.2.2 Data acquisition

For MIR analysis, potassium bromide (KBr) was selected as the window and sample matrix material, primarily due to its excellent infrared transmission properties and chemical stability. During sample preparation, rice powder samples were precisely mixed with dry KBr powder at a mass ratio of 1:100, thoroughly ground to a fine and uniform consistency, and then pressed into uniform, crack-free semi-transparent pellets under a pressure of 10 T·cm⁻² using a hydraulic press. For each origin, 100 spectra were collected. After quality assessment, 60 optimal spectra with higher SNR and stable signals were selected for subsequent analysis. Each spectrum represented the average result of 3 repeated scans per sample to enhance data reliability.

The KBr pellet method, through fine grinding and high-pressure forming, effectively ensured sample homogeneity and particle size consistency, significantly reducing light scattering interference, making it particularly suitable for quantitative analysis. Although Attenuated Total Reflectance (ATR) technology offers more convenient sample preparation, its signal intensity is susceptible to variations in sample-crystal contact conditions, potentially introducing variability. To maintain consistency and comparability with historical data, the classical KBr pellet method was chosen for this study. Subsequent research could consider introducing ATR technology and establishing corresponding spectral conversion models for method interoperability.

For FLU measurement, the light source was positioned 300 mm directly above the sample center, with the emission direction at a 45° angle to the horizontal plane to minimize surface reflection interference and optimize excitation efficiency. Each sample was measured 100 times in parallel. Strict screening based on SNR and signal intensity consistency was performed, ultimately retaining 60 representative spectra for statistical analysis, ensuring data quality met modeling requirements.

### 2.3 Spectral data processing methods

#### 2.3.1 Preprocessing methods

Factors such as instrument noise, baseline drift, and light scattering effects during spectral acquisition can significantly impact data quality. To improve model stability and reliability, this study employed a multi-step preprocessing approach to optimize MIR and FLU spectral data. Firstly, all raw spectral data underwent normalization (Normalization), linearly transforming spectral intensity values to the standard [0, 1] interval to eliminate effects from differences in sample concentration and measurement conditions (Flores-Morales et al., 2012).

To address random noise, the Savitzky-Golay (SG) convolution smoothing algorithm was applied. This algorithm effectively suppresses high-frequency noise while preserving spectral feature peak shapes. To eliminate light scattering effects, Multiplicative Scatter Correction (MSC) was specifically introduced. This method corrects for light scattering interference caused by uneven sample particles by establishing an ideal scatter model (Ye et al., 2018). The aforementioned preprocessing workflow significantly enhanced the SNR of the spectral data, providing a high-quality data foundation for subsequent modeling analysis.

#### 2.3.2 Feature selection method based on SPA

To address the susceptibility of high-dimensional spectral data to overfitting, this study adopted a systematic feature selection strategy. The feature wavelength screening method based on the Successive Projections Algorithm (SPA) employs vector projection principles for iterative calculation, progressively eliminating redundant and collinear wavelength variables, ultimately screening out a feature wavelength combination with minimal redundancy and maximum information content (Soares et al., 2013).

Regarding sample partitioning, the Kennard-Stone (KS) algorithm was used to achieve uniform distribution of the sample space. The dataset was divided into a training set (70%), a validation set (20%), and a test set (10%). The training set was used for model construction, the validation set for parameter tuning, and the test set for final performance evaluation.

### 2.4 Classification model methods

For classification model construction, this study employed three machine learning algorithms: Support Vector Machine (SVM), Gradient Boosting Decision Tree (GBDT), and Logistic Regression (LR) for model training and optimization.

#### 2.4.1 Model selection and parameter settings

1. The SVM model utilized the Radial Basis Function (RBF) kernel. Key parameters were optimized via grid search to balance model complexity and generalization capability.
2. The GBDT model was configured with a maximum decision tree depth of 5, a learning rate of 0.1, and 100 iterations. An early stopping mechanism was incorporated to prevent overfitting.
3. The LR model employed L2 regularization. The regularization coefficient C was determined through 5-fold cross-validation to ensure model stability.

#### 2.4.2 Model performance evaluation metrics

Accuracy, Precision, Recall, and F1-score were adopted as evaluation metrics for model performance. Given that this study involves a multi-class classification task (distinguishing 8 different origins), these metrics were calculated based on a “One-vs-Rest” (OvR) strategy. Specifically, for each origin, it was treated individually as the “positive class,” while samples from all other origins were collectively considered the “negative class.” Corresponding True Positives (TP), False Positives (FP), True Negatives (TN), and False Negatives (FN) were calculated for each class. The final reported Precision, Recall, and F1-score are Macro-average values, meaning the average of the individually calculated metric values for all 8 classes (Soares et al., 2013), ensuring equal weight for each origin regardless of sample size differences.

Four metrics were selected for comprehensive model performance evaluation:

1. Precision: Accuracy of positive predictions.
2. Recall: Identification rate of positive samples.
3. F1-score: Harmonic mean of Precision and Recall.

Model performance was comprehensively assessed using these four metrics. Calculation formulas are as follows:


$$\text{Accuracy}=\left(\text{TP}+\text{TN}\right)/\left(\text{TP}+\text{FP}+\text{TN}+\text{FN}\right);$$

$$\text{Precision}=\text{TP}/\left(\text{TP}+\text{FP}\right);$$

$$\text{Recall}=\text{TP}/\left(\text{TP}+\text{FN}\right);$$

$$F1-\text{score}=2\times \left(\text{Precision}\times \text{Recall}\right)/\left(\text{Precision}+\text{Recall}\right).$$

### 2.5 Data fusion technology

This study proposed an analysis method based on multi-source spectral data fusion, achieving synergistic analysis of MIR and FLU data through two technical routes: data-level fusion and feature-level fusion. Addressing the technical challenges of multi-instrument data integration, a preprocessing pipeline was established: Min-Max normalization was used to standardize spectral intensities to the [0,1] interval, eliminating the effects of concentration differences and measurement conditions.

In the data-level fusion strategy, the preprocessed MIR and FLU spectral matrices were concatenated along the wavelength dimension to construct a joint feature matrix [n × (p_MIR + p_FLU)], and the SPA was used to (screen for) the optimal feature combination. In the feature-level fusion strategy, SPA feature selection was first performed separately on the two types of spectra, and then the selected feature variables were concatenated to form a fused feature vector.

## 3 Results and discussion

Spectral image analysis revealed that although the overall spectral characteristics of rice from different origins were highly similar, significant differences emerged in specific wavelength regions. These differences reflect subtle variations in the structure and content of rice chemical components. Given that mid-infrared (MIR) spectroscopy excels at characterizing the molecular structure and abundance of major chemical components in rice, while fluorescence (FLU) spectroscopy provides precise insights into the electronic structure and concentration of specific fluorophores, their integration significantly enhances the accuracy of rice geographical origin traceability through a complementary analytical strategy.

### 3.1 Mid-infrared spectral data

#### 3.1.1 MIR spectral data preprocessing

**Figure 2:**
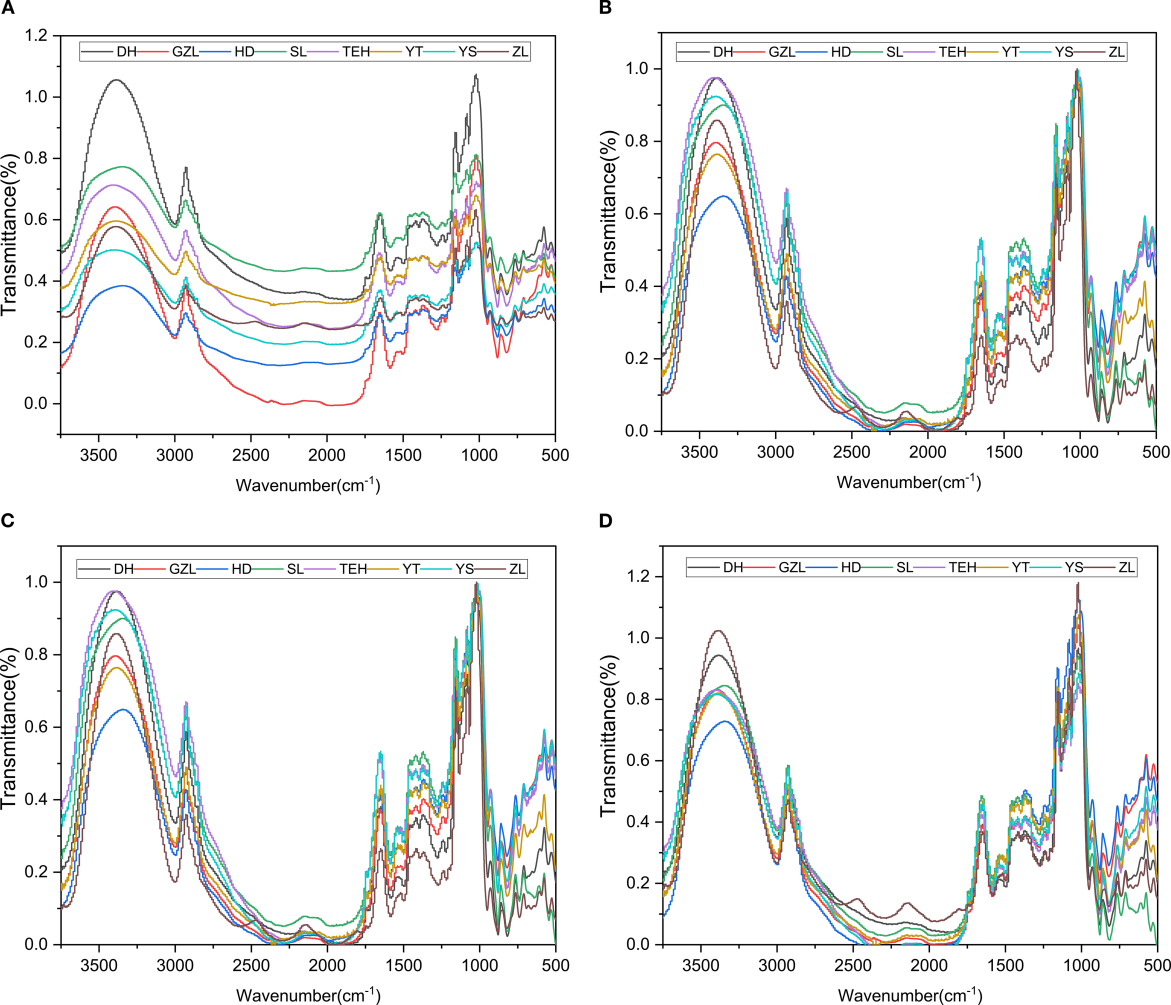
Average spectral preprocessing plots of rice powder MIR spectra. **(a)** Raw MIR spectra of rice powder; **(b)** Normalized MIR spectra of rice powder; **(c)** Normalization-SG processed MIR spectra of rice powder; **(d)** Normalization-MSC processed MIR spectra of rice powder.

To visually display the differences in rice spectral data from different origins, the average spectral data for rice samples from each origin were calculated, and a comparison chart of average spectral data between regions was plotted (see **Figure 2**). (**Figure 2a**) shows the raw MIR spectra of the samples, generated by molecular vibrational transitions. Given the significant noise in spectral data below 500 cm^-1^ and above 3750 cm^-1^, this study specifically selected the 500–3750 cm^-1^ band as effective spectral data for analysis, as detailed in **Figure 2**. Due to the complexity and severe overlap of bands in the IR region, preprocessing methods were employed to effectively extract sample information. Specific results are shown in (**Figure 2b**) Normalization, (**Figure 2c**) Normalization-SG smoothing filter, and (**Figure 2d**) Normalization-MSC scatter correction. Comparing (**Figure 2b**) with (**Figures 2c, d**), it can be observed that the spectra processed with smoothing and scatter correction are smoother than the original spectra, and the differences between the spectral curves are significantly reduced. This indicates that MSC preprocessing effectively corrected spectral errors caused by scattering phenomena during spectral acquisition.

#### 3.1.2 MIR Spectral analysis

**Figure 3:**
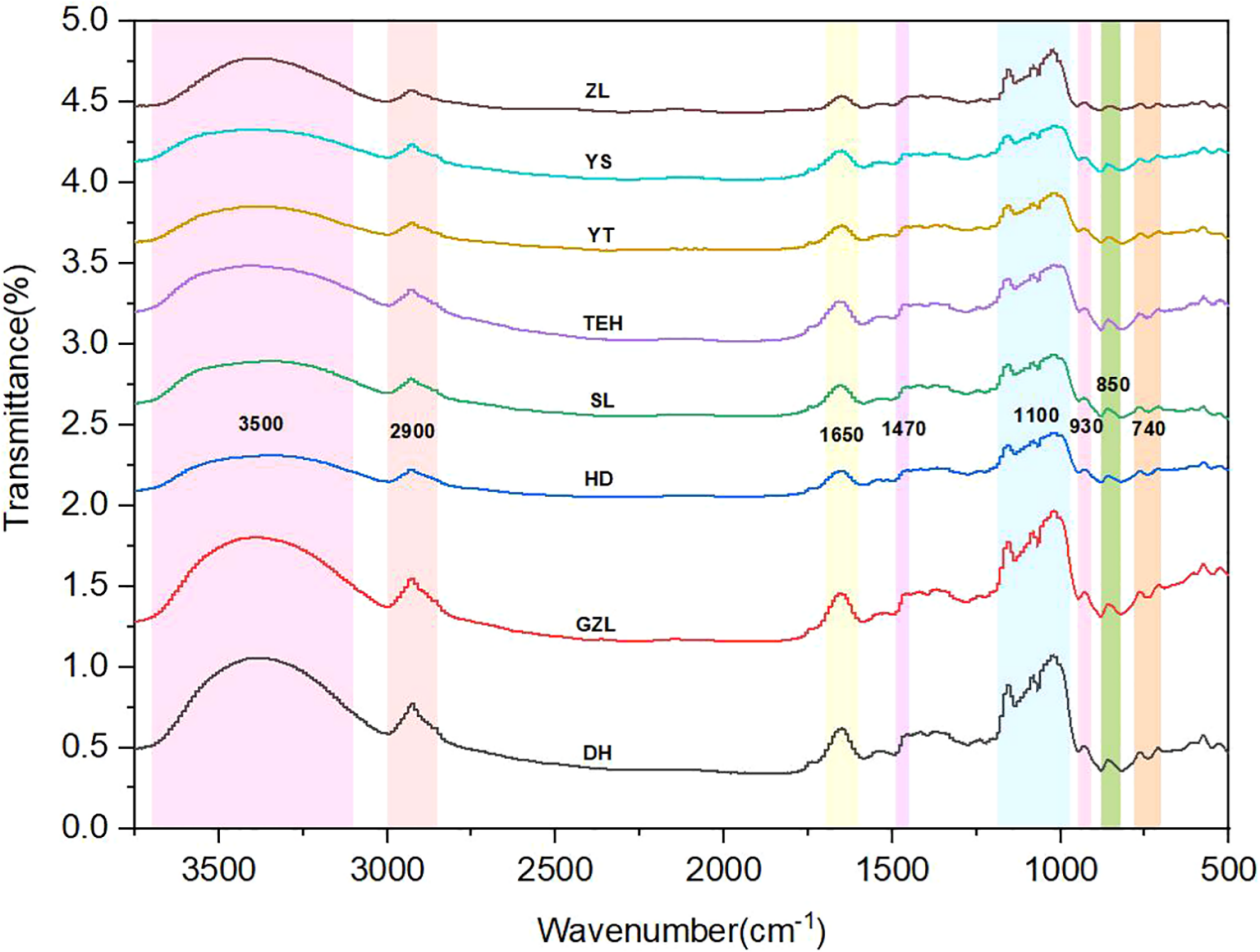
Assignment diagram of rice powder MIR spectra.

FTIR analysis indicated that rice powder displayed characteristic absorption peaks within the 500–3750 cm^-1^ range, closely related to molecular vibrations of its main components (starch, protein, lipids, and water molecules) (**Figure 3**). The specific assignments of the absorption peaks are as follows: The broad and strong absorption band observed in the 3700–3000 cm^-1^ range (centered around 3500 cm^-1^) is primarily attributed to the O-H stretching vibration of water molecules and the N-H stretching vibration of the protein amide A band. The characteristic peaks in the 3000–2800 cm^-1^ range (around 2900 cm^-1^) correspond to the asymmetric and symmetric C-H stretching vibrations of methylene (CH_2_) groups in lipids, indicating the presence of long-chain fatty acids. The characteristic peaks observed around 1600–1700 cm^-1^ and 1490–1450 cm^-1^ correspond to the protein amide I band (C=O stretching vibration) and amide II band (N-H bending vibration/C-N stretching vibration) (Zhang et al., 2023b), respectively. These peaks may overlap with the O-H bending vibration of starch. The strong absorption band in the 1190–950 cm^-1^ range is characteristic of starch, where the peak around 1150 cm^-1^ is assigned to C-O-C stretching vibration, and the multiple peaks in the 1000–1090 cm^-1^ range correspond to C-O-H stretching vibrations. These characteristic peaks are closely related to the crystallinity and molecular structure of starch (Lian et al., 2013). In the 900–500 cm^-1^ range, the characteristic peak around 950–910 cm^-1^ can be attributed to the ring vibration of the α-1,4 glycosidic bond in starch, while the weak peak around 760–700 cm^-1^ may be related to the skeletal vibrations of polysaccharides. **Table 2** summarizes the assignment results of the key absorption peaks in the mid-infrared spectrum of rice powder.

**Table 2:** Assignment of key absorption peaks in the MIR spectra of rice powder.

Wavenumber range (cm⁻¹)	Vibration type	Assigned compound/functional group
3700–3000	O-H stretching vibration	Water
3000–2800	C-H stretching vibration	Lipids
1600–1700	C=O stretching vibration	Proteins
1490–1450	C-H bending vibration	Lipids and proteins
1190–950	C-O-C/C-O stretching vibration	Starch
950–910	Ring vibration	Starch
880–820	C-C stretching vibration	Starch
760–700	Skeletal vibration	Polysaccharide ring structures

### 3.2 Fluorescence spectral data

#### 3.2.1 FLU spectral data preprocessing

**Figure 4:**
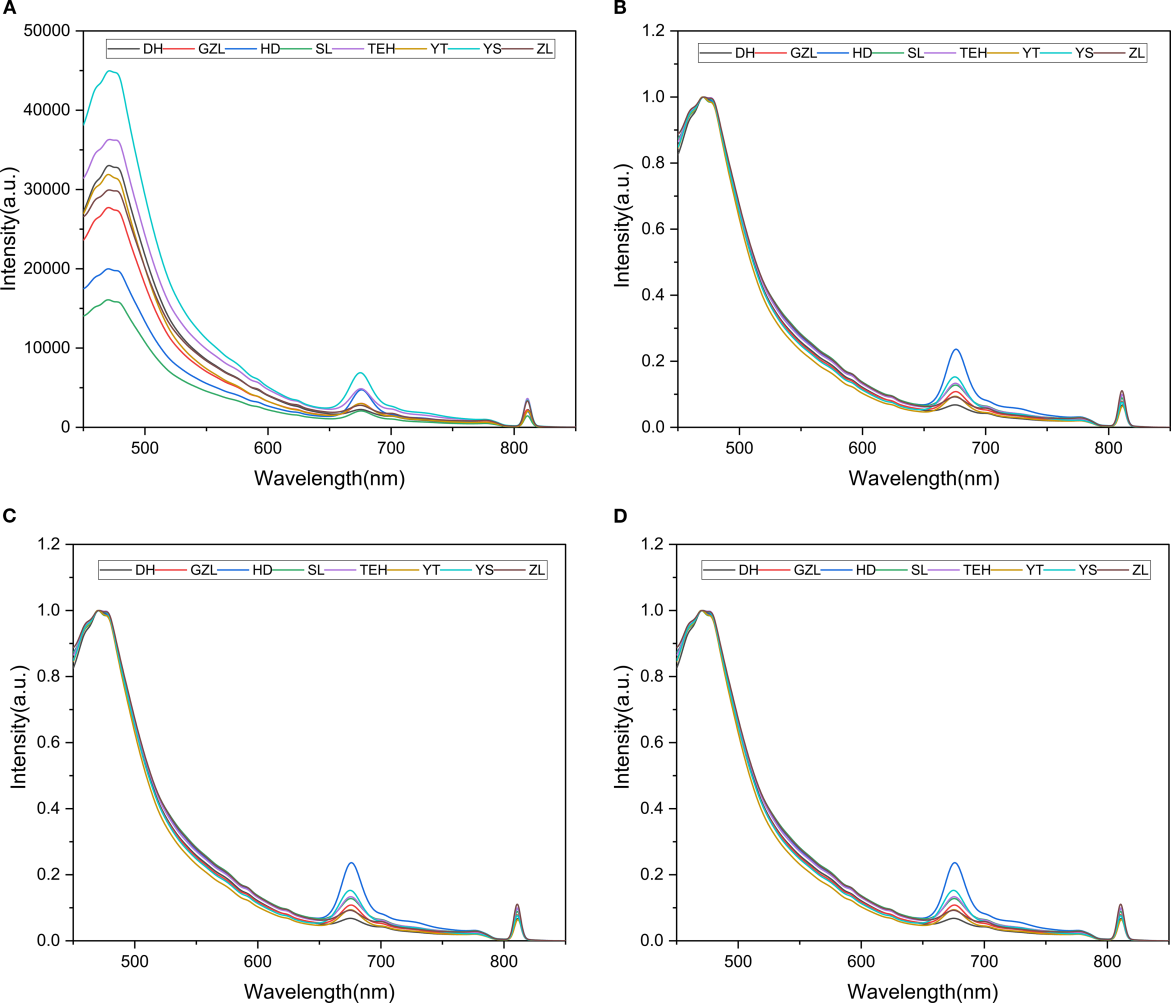
Preprocessing plots of rice powder FLU spectra. **(a)** Raw FLU spectra of rice powder; **(b)** Normalized FLU spectra of rice powder; **(c)** Normalization-SG processed FLU spectra of rice powder; **(d)** Normalization-MSC processed FLU spectra of rice powder.

To accurately characterize the spectral properties of rice, spectral data from rice of different origins were collected, and the characteristic spectral curve of rice was constructed based on the mean of these 100 data points. To improve data quality, denoising was performed, removing bands with significant noise such as 350–379 nm, and retaining the effective band from 433 nm to 800 nm, as shown in (**Figure 4a**). The intensity values of the F LU spectra were much greater than those of the MIR spectra. To eliminate the magnitude differences between the data, the FLU spectral data were normalized, as shown in (**Figure 4b**). Through MSC and SG preprocessing, data interference was significantly reduced, ensuring the accuracy of subsequent analysis. The results are shown in (**Figures 4c, d**).

The main components of rice, such as starch and protein, exhibit different vibrational spectral characteristics due to differences in chemical composition, content, and structure. Significant fluorescence characteristic peaks were observed particularly in the wavelength intervals of 475–525 nm, 550–600 nm, and 650–690 nm. During analysis, it cannot be assumed that a FLU spectral peak originates from a single substance solely based on its position, as some peaks may result from mixtures of multiple substances.

#### 3.2.2 FLU spectral analysis

**Figure 5:**
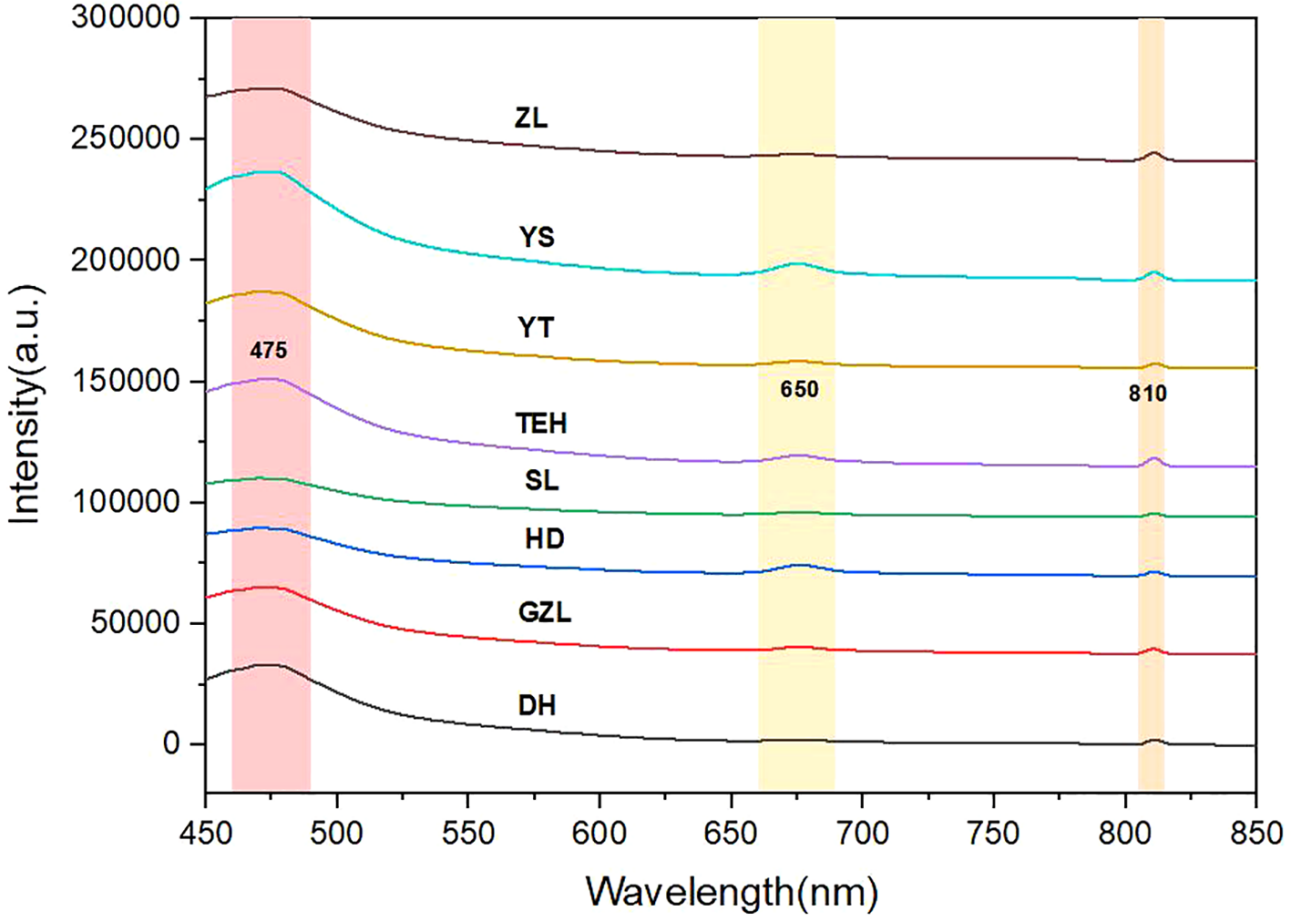
Assignment diagram of rice powder FLU spectra.

Under a 405 nm excitation wavelength, the FLU spectra (450–850 nm) of rice powder displayed typical fluorescence characteristics (**Figure 5**). The strong fluorescence emission peak in the 460–490 nm range (maximum emission ~495 nm) is primarily attributed to phenolic compounds in rice. The FLU signal detected in the 660–690 nm range may originate from protein-pigment complexes or lipid oxidation products.

#### 3.2.3 Feature band selection results

The modeling in this study is based on a total of 480 spectral samples covering 8 different origins. To address the high-dimensional data with up to 7,625 bands, the authors employed the Successive Projections Algorithm (SPA) for feature wavelength selection, with key parameters set as follows: minimum variables (MinVariable) = 200, maximum variables (MaxVariable) = 500, and cross-validation was used for evaluation.

**Figure 6:**
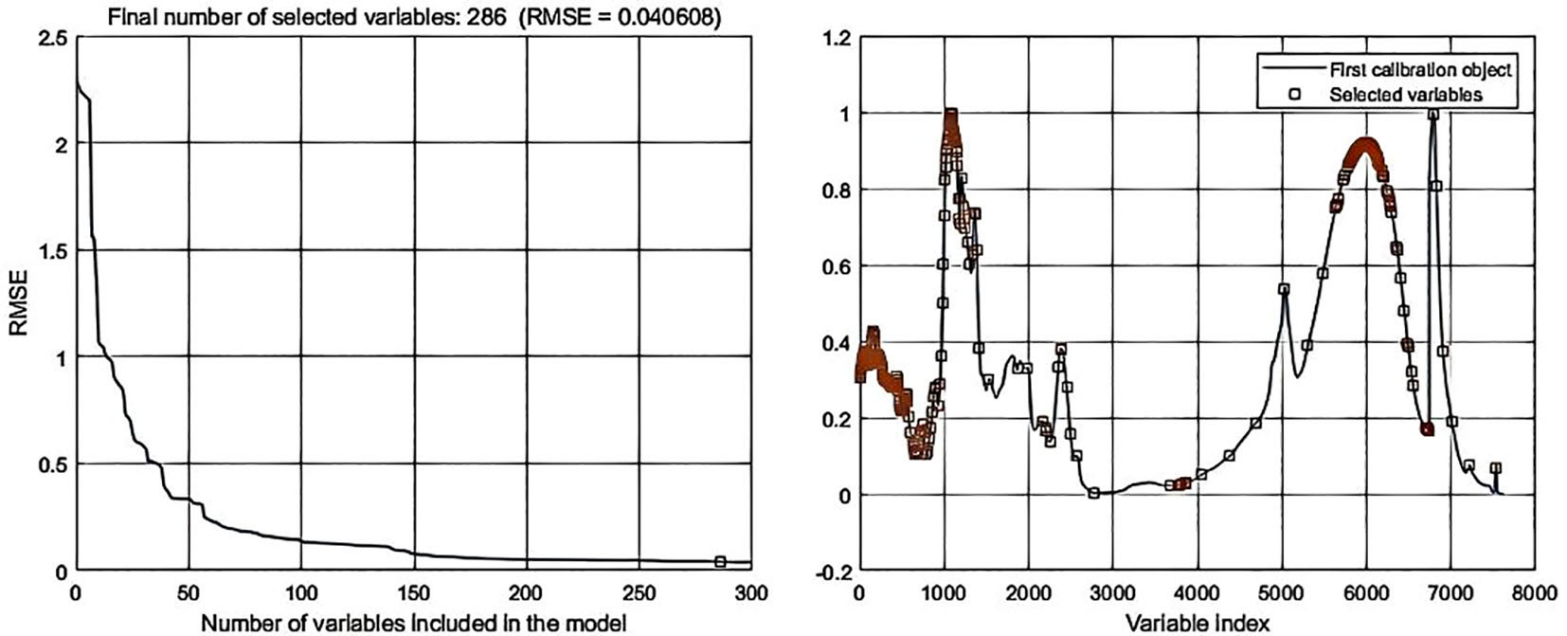
SPA error analysis and feature selection distribution for data-level fusion with Normalization-SG preprocessing.

**Figure 7:**
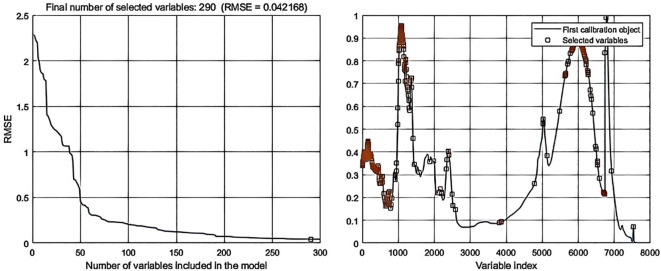
SPA error analysis and feature selection distribution for data-level fusion with Normalization-MSC preprocessing.

**Figure 8:**
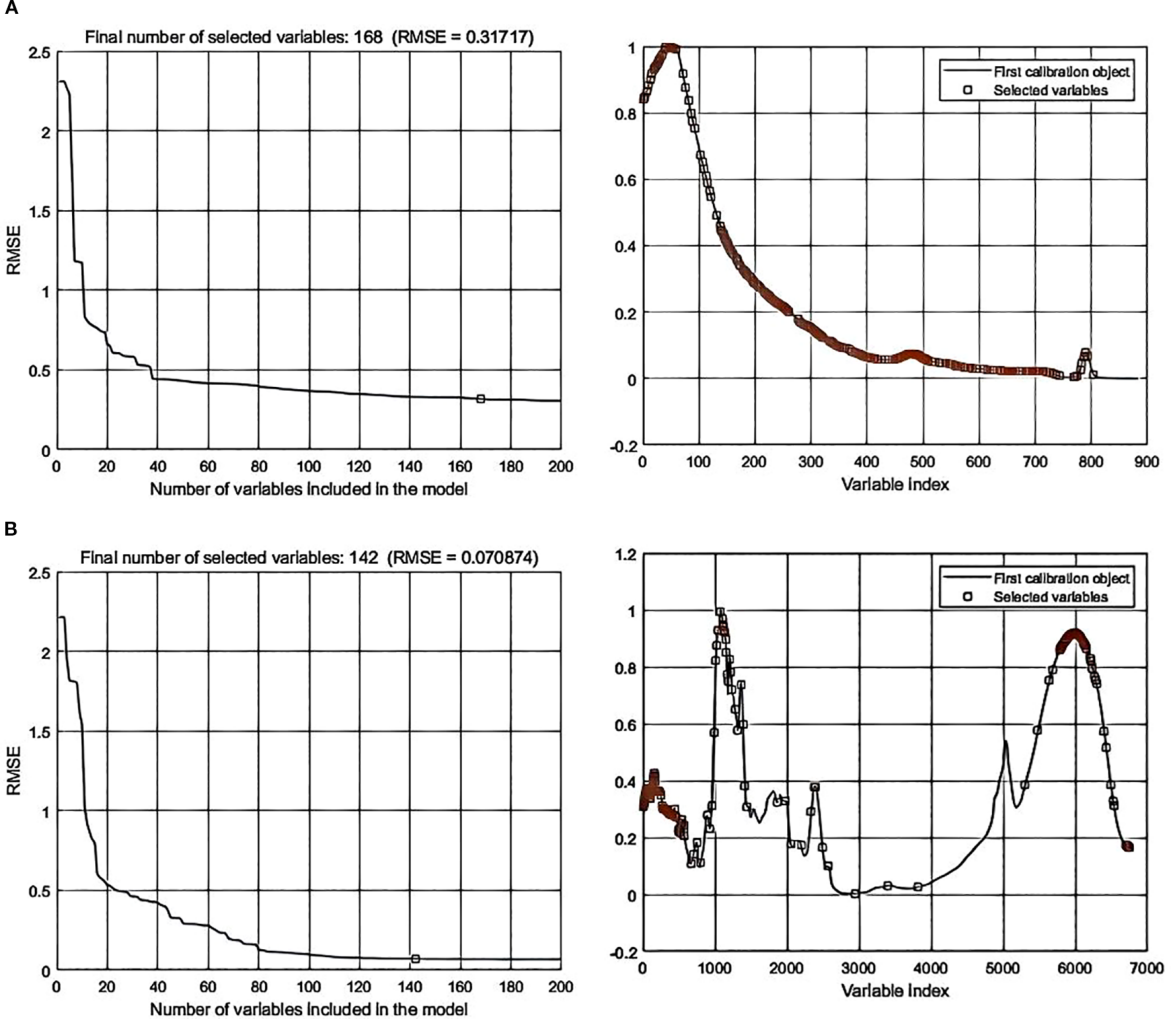
SPA error analysis and feature selection distribution for feature-level fusion with Normalization-SG preprocessing. **(a)** Fluorescence spectra; **(b)** Mid-infrared spectra.

**Figure 9:**
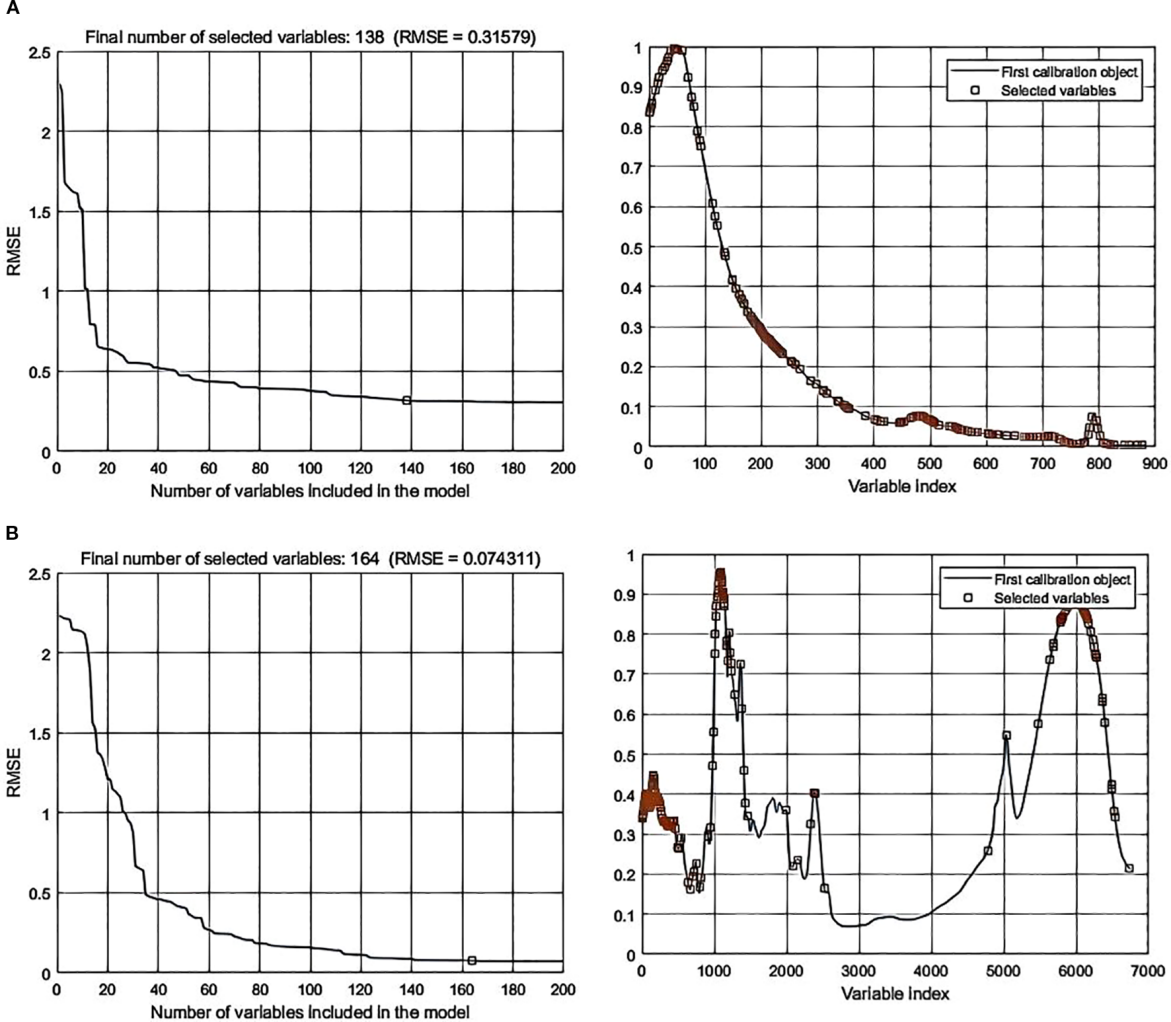
SPA error analysis and feature selection distribution for feature-level fusion with Normalization-MSC preprocessing. **(a)** Fluorescence spectra; **(b)** Mid-infrared spectra.

Processing the spectral data through SPA error analysis and feature selection distribution yielded the following results: In data-level fusion, the Normalization-SG preprocessing method selected 286 features (**Figure 6**), and the Normalization-MSC method selected 290 features (**Figure 7**). In feature-level fusion, the Normalization-SG method selected a total of 310 features [168 FLU features (**Figure 8a**) and 142 MIR features (**Figure 8b**)], while the Normalization-MSC method selected 302 features [138 FLU features (**Figure 9a**) and 164 MIR features (**Figure 9b**)]. Compared to the original unprocessed spectra dimensionality of 7625 (None), this method achieved significant data dimensionality reduction. As shown in **Table 3**, this feature selection strategy not only effectively reduced data dimensionality but also significantly improved the model’s generalization performance, providing an optimized feature selection solution for both data-level and feature-level fusion.

**Table 3:** Comparison of feature selection results for data-level and feature-level fusion based on the SPA algorithm.

Fusion strategy	Preprocessing method	
	Normalization-SG	Normalization-MSC
None	7625	7625
Data-Level Fusion Feature-Level Fusion	286	290
Data-Level Fusion Feature-Level Fusion	310	302

#### 3.2.4 Selection of processing methods

This study systematically compared the effects of the Normalization, Normalization-SG (Savitzky-Golay smoothing normalization), and Normalization-MSC (multiplicative scatter correction normalization) preprocessing methods on fused rice spectral data across two dimensions: data-level fusion and feature-level fusion. By comprehensively evaluating the performance of each preprocessing method, the optimal preprocessing scheme was screened out for constructing efficient origin discrimination models.

During the model building process, the preprocessed spectral data served as input features for a Random Forest (RF) classifier. Simultaneously, a multi-dimensional model performance evaluation system was constructed using three metrics: Precision, Recall, and F1-score. The increase in evaluation metric values showed a significant positive correlation with the optimization of preprocessing effects; that is, higher metric values indicated more reasonable preprocessing method selection and superior performance of the constructed origin discrimination model.

Experimental results revealed notable differences in optimal preprocessing methods under different fusion strategies (**Table 4**). For data-level fusion, both Normalization-SG and Normalization-MSC achieved excellent training performance (Precision > 0.99). However, on the test set, Normalization-SG significantly outperformed Normalization-MSC across all metrics (Precision: 0.9095 vs. 0.8832; Recall: 0.9062 vs. 0.8750; F1-score: 0.9064 vs. 0.8730).

**Table 4:** Comparison of Random Forest modeling results under data-level and feature-level fusion.

No.	Fusion strategy	Preprocessing method	Training set			Test set		
			Precision	Recall	F1-Score	Precision	Recall	F1-Score
1	Data-level fusion	Normalization-SG	0.9820	0.9817	0.9818	0.9095	0.9062	0.9064
		Normalization-MSC	0.9654	0.9635	0.9631	0.8832	0.8750	0.8730
		Normalization	0.9818	0.9817	0.9817	0.9392	0.9375	0.9370
2	Feature-level Fusion	Normalization-SG	0.9950	0.9947	0.9947	0.9527	0.9479	0.9470
		Normalization-MSC	0.9926	0.9921	0.9922	0.9268	0.9270	0.9244
		Normalization	0.9923	0.9921	0.9921	0.9431	0.9375	0.9366

This advantage was even more pronounced in feature-level fusion. The performance metrics of Normalization-SG on the test set (Precision: 0.9527, Recall: 0.9479, F1-score: 0.9470) were approximately 2.6%–2.9% higher than those of Normalization-MSC (Precision: 0.9268, Recall: 0.9270, F1-score: 0.9244). Notably, Normalization-SG demonstrated stronger generalization capability on the test set while maintaining high accuracy on the training set (Data-level: 0.9820; Feature-level: 0.9950), indicating that its preprocessing strategy more effectively preserves the discriminative features of the data, thereby significantly enhancing model robustness across different fusion scenario.

### 3.3 Establishment and analysis of the data fusion discrimination model

This study systematically compared the performance of different data fusion strategies combined with machine learning algorithms for the rice origin discrimination task. The experiment employed two fusion strategies: feature-level fusion and data-level fusion, uniformly applying the Normalization-SG method for spectral data preprocessing. Evaluated algorithms included Support Vector Machine (SVM), Gradient Boosting Decision Tree (GBDT), and Logistic Regression (LR).

The specific parameter configurations for each model were as follows. For the SVM model, the Radial Basis Function (RBF) kernel was utilized, with the gamma parameter set to ‘scale’ and the regularization parameter C set to 1.0. This configuration adheres to the default settings in scikit-learn and, although not systematically optimized, provides a balance of good generality and reproducibility. In the case of the GBDT model, the number of estimators (n_estimators) was set to 80 and the learning rate to 0.1, a configuration determined through preliminary experiments to achieve an effective trade-off between predictive performance and computational efficiency. Early stopping was enabled during training with n_iter_no_change = 10 and tol = 1×10⁻^4^, which resulted in the actual training terminating early at the 72nd iteration. For the LR model, the maximum number of iterations (max_iter) was set to 100, with the ‘saga’ solver selected and elastic net regularization (l1_ratio = 0.5) applied. This setup integrates the benefits of both L1 and L2 regularization, facilitating feature selection and improving handling of multicollinearity, while the chosen solver and iteration count ensured stable convergence during optimization.

**Figure 10:**
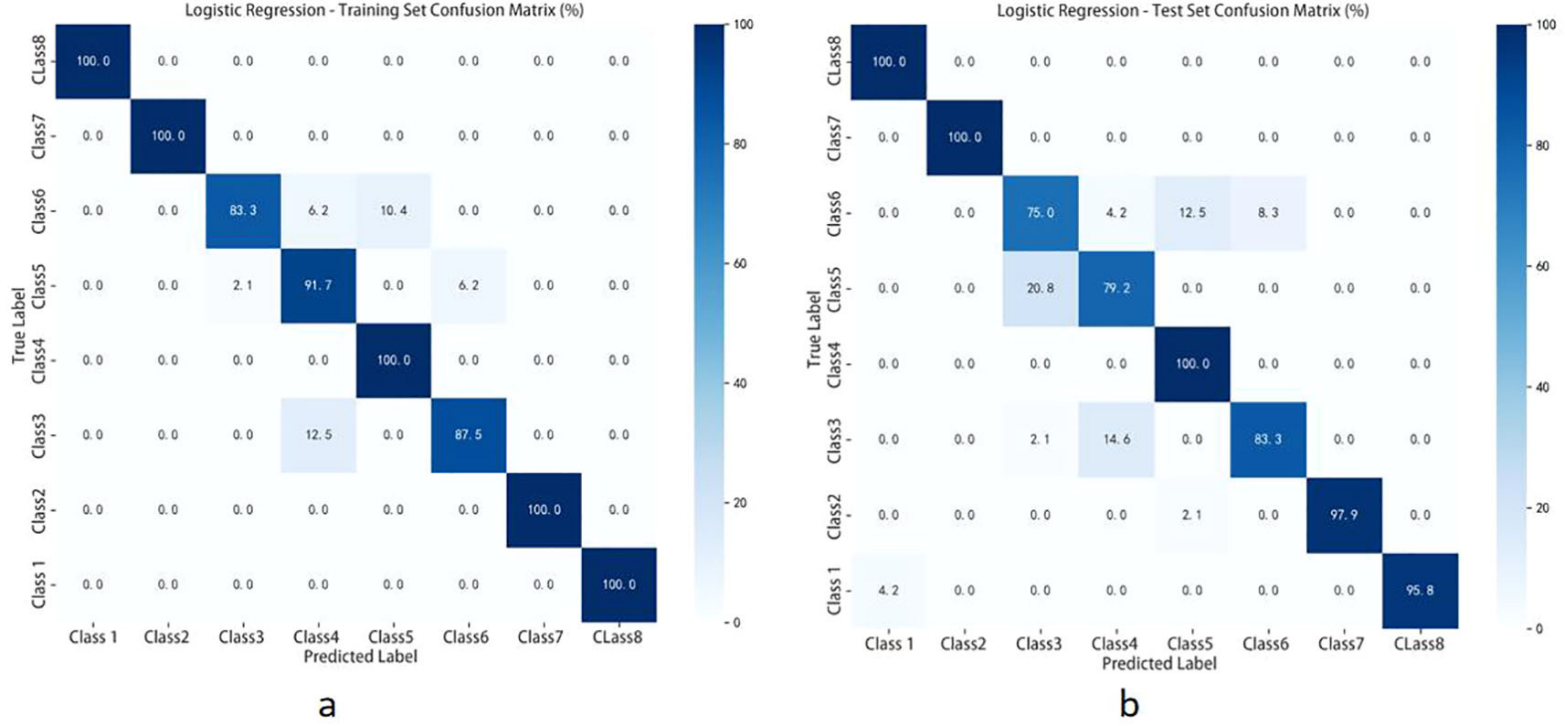
Shows the confusion matrix results of the test set: **(a)** classification performance of the logistic regression (LR) model under data-level fusion mode; **(b)** classification performance of the logistic regression (LR) model under feature-level fusion mode.

The performance of different fusion strategy and algorithm combinations is comprehensively compared in **Table 5**, while the classification results of the Logistic Regression (LR) model on the test set are further illustrated via the confusion matrix in **Figure 10**. The main findings indicate that under data-level fusion, the LR model integrated with Normalization-SG preprocessing achieved the highest average classification accuracy of 91.45%. Similarly, under feature-level fusion, the same preprocessing approach combined with LR again delivered optimal performance, attaining an average accuracy of 95.55%, significantly surpassing other algorithms. Overall, feature-level fusion consistently exceeded data-level fusion across most evaluation metrics, demonstrating its enhanced capability to retain discriminative features and minimize information redundancy. Although the Gradient Boosting Decision Tree (GBDT) model reached 100% accuracy on the training set, evident overfitting led to its exclusion from primary comparative analysis. The Support Vector Machine (SVM) exhibited consistent performance under both fusion strategies, yet was consistently outperformed by LR. These outcomes underscore the crucial impact of fusion strategy and algorithm selection on the efficacy of spectral data-based origin identification, with the combination of feature-level fusion and LR emerging as the most promising approach.

**Table 5:** Comparison of modeling results for data-level and feature-level fusion strategies.

No.	Fusion strategy	Spectral dimension	Modeling method	Training set			Test set		
				Precision	Recall	F1-Score	Precision	Recall	F1-Score
1	Feature-Level	310	Logistic Regression	0.9555	0.9531	0.9529	0.9305	0.9166	0.9181
			SVM	0.8015	0.7812	0.7798	0.8317	0.8125	0.8093
			Gradient Boosting	1	1	1	0.9615	0.9583	0.9586
2	Data-Level Fusion	286	Logistic Regression	0.9145	0.9140	0.9134	0.8884	0.8750	0.8755
			SVM	0.8348	0.8072	0.7971	0.8014	0.7604	0.7409
			Gradient Boosting	1	1	1	0.9546	0.9479	0.9476
3	Fusion without feature selection	7625	Logistic Regression	1	1	1	0.9694	0.9687	0.9686
			SVM	0.8381	0.8385	0.8360	0.8044	0.7812	0.7757
			Gradient Boosting	1	1	1	0.9267	0.9270	0.9261
4	Mid-Infrared Spectroscopy	6742	Logistic Regression	1	1	1	0.9694	0.9687	0.9686
			SVM	0.8527	0.8359	0.8338	0.8044	0.78125	0.775
			Gradient Boosting	1	1	1	0.9151	0.9062	0.9044
5	Fluorescence Spectroscopy	883	Logistic Regression	0.9948	0.9947	0.9947	0.9149	0.9166	0.9132
			SVM	0.8835	0.8906	0.8760	0.8721	0.875	0.8681
			Gradient Boosting	1	1	1	0.8268	0.8020	0.7968

### 3.4 Discussion

Based on the experimental results, the following analyses can be drawn:

#### 3.4.1 Effectiveness of feature-level fusion

Feature-level fusion generally performed better than data-level fusion, consistent with conclusions from existing multi-source spectral data fusion research. This indicates that fusion at the feature extraction stage is more conducive to extracting cross-source discriminant features.

Importance of Preprocessing Methods: The effectiveness of the Normalization-SG method in processing spectral data was validated. It significantly enhanced model generalization ability, aligning with conclusions from preprocessing strategies in related Raman spectroscopy studies.

#### 3.4.2 Overfitting issue

GBDT’s perfect performance on the training set but fluctuating performance on the test set indicated overfitting. Therefore, complex ensemble methods should be used cautiously, or stronger regularization should be introduced when emphasizing generalization capability.

Potential of High-Dimensional Fusion: Although the “Fusion (No Feature Selection)” approach had an extremely high dimensionality (7625), LR still performed well, suggesting the raw data contains substantial effective information. This implies that combining dynamic feature selection could further enhance model performance and efficiency.

#### 3.4.3 Model robustness comparison

LR demonstrated robustness and accurate classification in both fusion modes, making it particularly suitable for medium-to-low dimensional spectral data. SVM was more sensitive to parameters and feature scaling, and its performance was not fully realized without parameter tuning.

The results of this study indicate that strategies based on spectral data fusion can be effectively used for rice origin discrimination. Among them, the combination of feature-level fusion, LR, and Normalization-SG preprocessing constituted the optimal model configuration under the experimental conditions of this study, offering both high classification accuracy and good stability. Future research could introduce dynamic feature selection and weighted fusion strategies to further improve model performance and generalization capability in complex origin discrimination tasks.

## 4 Conclusions

This study systematically established a rice origin identification method based on multispectral fusion, yielding the following conclusions: (1) The feature-level fusion strategy outperforms data-level fusion, achieving a classification accuracy of 95.55%, indicating that the approach of feature selection followed by fusion better preserves effective information; (2) The Normalization-SG preprocessing combination performed best, with a test set F1 score of 0.9470, confirming its advantages in feature retention and noise suppression; (3) The logistic regression algorithm achieved the best balance between accuracy (93.05%) and robustness, making it suitable for practical applications; (4) SPA feature selection reduced data dimensionality by over 96%, significantly improving model efficiency. The innovation of this study lies in establishing a standardized multispectral fusion analysis process, addressing technical challenges in data integration.

Experimental results demonstrate that mid-infrared spectroscopy (500–3750 cm-1) and fluorescence spectroscopy (450–850 nm) exhibit significant complementarity, and their synergistic analysis can comprehensively characterize the chemical composition characteristics of rice. This method not only achieves high classification accuracy but also is simple to operate, reproducible, and has promising prospects for promotion and application.

Although this study focused on specific rice varieties, the established analytical framework demonstrates strong generalizability and extensibility. Future work will validate its applicability to a wider range of cultivars and geographical regions. The proposed methodology can also serve as a technical paradigm and successful practice for traceability studies of other high-value agricultural products.

## Data Availability

The original contributions presented in the study are included in the article/supplementary material. Further inquiries can be directed to the corresponding author.
